# Motor imagery ability in stroke patients: the relationship between implicit and explicit motor imagery measures

**DOI:** 10.3389/fnhum.2013.00790

**Published:** 2013-11-19

**Authors:** Sjoerd de Vries, Marga Tepper, Wya Feenstra, Hanneke Oosterveld, Anne M. Boonstra, Bert Otten

**Affiliations:** ^1^Centre for Human Movement Sciences, University Medical Centre Groningen, University of GroningenGroningen, Netherlands; ^2^Research Centre for Health, Social Work & Technology, School of Applied Psychology, Saxion University of Applied SciencesDeventer, Netherlands; ^3^Department of Rehabilitation Medicine, University Medical Centre GroningenGroningen, Netherlands; ^4^‘Revalidatie Friesland’ Centre for RehabilitationBeetsterzwaag, Netherlands

**Keywords:** motor imagery, rehabilitation, hand laterality, phenomenology, questionnaire, implicit, stroke

## Abstract

There is little consensus on how motor imagery ability should be measured in stroke patients. In particular it is unclear how two methods tapping different aspects of the motor imagery process relate to each other. The aim of this study was to investigate the relationship between implicit and explicit motor imagery ability by comparing performance of stroke patients and controls on a motor imagery questionnaire and a hand laterality judgment task (HLJT). Sixteen ischemic stroke patients (36 ± 13 weeks post-stroke) and 16 controls, matched by age (51 ± 10 years), gender (7 females) and handedness (3 left-handed), performed a HLJT and completed a motor imagery questionnaire. Our study shows that neither in the healthy controls nor in patients, a correlation is found between the HLJT and the motor imagery questionnaire. Although the patient group scored significantly lower than the control group on the visual motor imagery component (*U* = 60; *p* = 0.010) and the kinesthetic motor imagery component (*U* = 63.5; *p* = 0.015) of the questionnaire, there were no significant differences between patients and controls on accuracy scores of the HLJT. Analyses of the reaction time profiles of patients and controls showed that patient were still able to use an implicit motor imagery strategy in the HLJT task. Our results show that after stroke performance on tests that measure two different aspects of motor imagery ability, e.g., implicit and explicit motor imagery, can be differently affected. These results articulate the complex relation phenomenological experience and the different components of motor imagery have and caution the use of one tool as an instrument for use in screening, selecting and monitoring stroke patients in rehabilitation settings.

## Introduction

The ability to imagine or simulate experiences is one of the most extraordinary capabilities of our mind. At first glance, certainly when our brain is intact, we do not realize that this capacity is more complex than the single homogenous capacity of imagery which we experience. Several studies have shown that mental imagery is a multifaceted capacity involving a number of different cognitive functions and brain areas (for a review see Kosslyn et al., 2001). Visual and motor imagery for instance are known to be linked with different neuronal subsystems and there is ample evidence of individual differences in imagery ability (Galton, 1883; Kosslyn, 1980; Richardson, 1994; Borst and Kosslyn, 2010). The present study addresses a specific question relating to measuring motor imagery ability in stroke patients, namely the question of how measures of implicit and explicit motor imagery relate to each other.

The use and explanation of the term motor imagery has led to substantial confusion about what the exact definition of motor, kinesthetic and visual imagery is and how these different perspectives are used by participants in practice. Classically motor imagery was defined as either from an internal, first person, perspective (as if someone was actually performing the imagined movement) or an external, third person, perspective (as if someone watched himself making the movement form outside of his body). Considerable confusion arose whether internal kinesthetic imagery should be dissociated from internal visual imagery (see also Hardy, 1997; Roberts et al., 2008 for a discussion). More recently, several researcher have concluded, independently, that the kinesthetic and first person internal perspective are best measured with a separate subscale in self-report questionnaires, reflecting a consensus on a more differentiated approach to measuring motor imagery ability (Roberts et al., 2008; Williams et al., 2012).

The debate leading to the confusion focused for a large part on the specific modalities in which movements could be imagined. According to Moran et al. (2012) some researchers appear to regard the use of the term kinesthetic motor imagery synonymous with an internal perspective whereas others have shown that the kinesthetic modality could also be experienced concurrently with the use of a third person, external, perspective (Hardy and Callow, 1999; Callow and Hardy, 2004). This latter position is in close accordance with neuroscience research showing that although different networks in the brain are involved with kinesthetic and visual imagery (see Jeannerod, 2001; Kosslyn et al., 2001 for a discussion) that these networks can also be activated simultaneously and are inherently tied to each other (Klatzky, 1994).

Jeannerod, in an influential account on the organization of action control in the brain, argued that representations that are used in the control of motor functions were also used in other cognitive domains, referring to motor imagery as a covert stage of action control (Jeannerod, 1994). Jeannerod and Frak defined motor imagery as “a subliminal activation of the motor system, a system which appears to be involved, not only in producing movements, but also in imagining actions, recognizing tools, learning by observation or even understanding the behavior of other people” (Jeannerod and Frak, 1999). More in particular, Jeannerod made a distinction between implicit and explicit motor imagery. He defined explicit motor imagery as the phenomenological experience where the feeling of the movement was experienced consciously. Explicit motor imagery ability is often measured with an introspective self-report such as the VMIQ-2, the KVIQ and the MIQ-R, where the vividness, clarity or easy with which the imagery is experienced is rated (Malouin et al., 2007; Roberts et al., 2008; Gregg et al., 2010). In contrast with explicit motor imagery, Jeannerod argued that motor imagery is also used implicitly. Here the representations of the motor system are used covertly, without awareness. Task relying on perceptually driven motor decisions, for instance judging whether a depicted hand is a left or a right hand, rely on the covert use of motor images. Also, prospective action judgments, for instance judging whether a dowel is graspable with a particular grip style, are examples of tasks relying on an implicit use of the motor system.

Characteristic of both forms of motor imagery in this account is the fact that the neural structures underpinning motor imagery share a remarkable neurological similarity with neural activity during movement execution. First, several studies showed that during imagination of a movement, more or less the same brain areas are involved as during the actual execution of a movement (Grèzes and Decety, 2001; Jeannerod, 2001; de Lange et al., 2005, 2008). Another similarity between execution and imagery of a movement is the equality in the time needed to mentally and physically perform the same movement (Decety and Jeannerod, 1995), a phenomenon known as mental isochrony. Further, there are strikingly similar physiological changes during movement imagination and actual performance (Guillot and Collet, 2005).

Interestingly, research into both forms of motor imagery, has shown very similar evidence for this covert use of the motor system in motor imagery. For instance, when asked to imagine walking between two points, the duration of the imagined walk is similar to time it would take to actual walk the same distance (Decety et al., 1989), and also shows a similar activity in neural areas used for motor planning and control (Roth et al., 1996). In the same regard, Parsons showed, when perceptually driven motor decisions are made whether a depicted hand is a left or a right hand [the hand laterality judgment task (HLJT)], that the time to judge whether the depicted hand is a left or right hand corresponds to the time it would take to actually perform a rotation of the arm and wrist in the orientation of the depicted hand (Parsons, 1987). Moreover, reaction times also corresponded with the awkwardness of the movement, biomechanical awkward movements took longer to complete, and several researchers showed corresponding activity in the motor areas of the brain when solving the HLJT (Parsons et al., 1995; Vingerhoets et al., 2002). These studies show that the biomechanical constraints and kinematic characteristics of actual movement are reflected in the performance of both implicit and explicit motor imagery measures. Therefore, it appears that implicit and explicit motor imagery are supported by motor representations of the motor system and that these processes seem to rely, at least partly, on equivalent underpinning mechanisms.

There is also long known neuropsychological evidence showing a dissociation between what can be consciously perceived and visual-motor abilities after lesions (see Willingham, 1998 for a discussion). For instance, in the visual-spatial domain, Milner and Goodale (1995) studied a patient who had limited conscious awareness of objects and was unable to recognize everyday objects or identify simple shapes visually. The same patient showed normal visual-motor abilities. The patient was perfectly able to orient a postcard correctly in line with a slit and was able to position the hand and fingers optimally for grasping objects. Examples of patients like the patient described by Milner and Goodale are also part of an on-going discussion about the boundaries between implicit and explicit memory. There is a large body of evidence showing that there are substantial differences between implicit and explicit learning and that these processes rely on different brain systems, although evidence of a partly common mechanism also exist (Dew and Cabeza, 2011). Moreover, it is now evident that motor skills can be learned implicitly (Masters, 1992; Jiménez and Méndez, 1999, 2001; Maxwell et al., 2003), even after stroke (Pohl et al., 2001; Orrell et al., 2006) without being consciously aware of what is learned. Moreover, in the domain of visual imagery an interesting study of a patient is described by Zeman et al. (2010) who rated himself as having almost no subjective visual imagery experiences.

These findings are relevant for recent developments in the field of rehabilitation. In the past decades motor imagery is increasingly been recommended as an additional technique that can be used for motor recovery in stroke rehabilitation (Jackson et al., 2001; Sharma et al., 2006; de Vries and Mulder, 2007). Herein, there has recently been an advocacy for the use of instruments to assess motor imagery ability of patients before they enter rehabilitation programs where motor imagery is used (Jackson et al., 2001; Braun et al., 2006, 2013 in this issue; de Vries and Mulder, 2007; Simmons et al., 2008) And, although a recent review has shown that a large number of studies still do not use motor imagery ability measures (Malouin et al., 2013 in this issue) a number of them do and they include both implicit and as well as explicit measures and there are also advances in developing instruments specific for the use in rehabilitation settings (Malouin et al., 2007, 2009; Gregg et al., 2010; Butler et al., 2012). However, there has been surprisingly little research about how self-report questionnaires that assess the vividness or ease of motor imagery ability relate to measures of implicit motor imagery when administered to stroke patients. To date, there is no consensus which instruments should be part of a motor imagery ability assessment and researchers use different instruments for screening purposes. In some studies implicit measures are used, others use only self-report questionnaires whereas others use a mix of different methods for assessing motor imagery ability. Given that lesions to the action system can affect implicit and explicit cognitive processing differently (see Willingham, 1998; Dew and Cabeza, 2011 for a discussion) shows that it is important to establish what the relationship between measures for implicit motor imagery ability and self-report rating is, particularly in the stroke population.

A recent study by McAvinue and Robertson (2007) with young healthy adults using a large test battery of motor and visual imagery measures is one of the few studies which shed some light on this issue. They showed that self-report ratings of motor imagery ability and tests that measure implicit motor imagery ability loaded onto different components, suggesting (in accordance with Jeannerod) an implicit and explicit component for motor imagery. In the same regard, Collet et al. (2011) have noticed that individual performance on different measures for motor imagery could vary and have made the suggestion that motor imagery ability might be best measured by using a combination score of different measures. However, these were studies including healthy adults which makes it difficult to generalize these findings to the stroke population. A study on stroke patients by Schwoebel and Coslett (2005) did also show evidence for a double dissociation between measures that require implicit judgments and measures that require explicit judgments. However, Schwoebel and Coslett did not include self-report ratings of the subjective experience of motor imagery in their study.

Given the lack of studies in stroke patients on the relationship between explicit self—report ratings and implicit measures of motor imagery ability it is unclear how they relate to each other. It could be that when a patient is impaired on one measure that he is also likely impaired on the other. However, the research by McAvinue and Robertson (2007) and Schwoebel and Coslett (2005) suggest that this need not be the case. Also, the study of the patient described Zeman et al. (2010) showed that, in the domain of visual imagery, the subjective experience of visual imagery could be absent after brain injury. A better insight in how implicit and subjective experience of explicit motor imagery relate to each other could contribute to a consensus on how motor imagery ability instruments can be used in the rehabilitation practice. Therefore, the aim of this study was to explore the prevalence of implicit and explicit motor imagery ability impairments and investigate the relationship between implicit and explicit motor imagery ability by comparing performance of stroke patients and controls on two different methods tapping both aspects of motor imagery.

Phenomenological self-report ratings that measure the vividness or ease of motor imagery ability require a conscious explicit judgment. Therefore, we used a motor imagery questionnaire, the MIQ-RS (Gregg et al., 2010), for measuring explicit motor imagery ability. A second method, perceptually driven motor decision tasks requires an unconscious judgment from the participant. Therefore, the HLJT was used for measuring implicit motor imagery ability (Parsons, 1987). We used the MIQ-RS because of the focus in this scale on items related to hand functioning. Thereby maximizing it's similarity with the HLJT. A simple choice reaction time task and a visual mental rotation task were used to control for non-motor-imagery specific cognitive impairments. With this setup we wanted to study what the prevalence of impairments on these instruments was and study the relationship between these measures of motor imagery ability is in stroke patients. Based on the results of the neuropsychological studies showing a dissociation between implicit and explicit cognitive processes and the study of McAvinue and Robertson (2007) where in healthy adults no correlation between the measures for implicit and explicit motor imagery were found we expected that patients would perform more poorly than controls on motor imagery tests but that conscious awareness of the ease with which a movement is imagined does not have to correlate with implicit motor imagery ability.

## Materials and methods

### Participants

In total sixteen patients (7 female, mean age 51.06 ± 10.74 years, 3 left-handed, 7 right hemisphere lesions) recovering from an ischemic stroke (36 ± 13 weeks ago) participated on voluntary basis in this the study. The participants took part in this study as part of a longitudinal study on monitoring motor imagery ability in stroke patients. Patients were recruited from stroke units of two rehabilitation centers in the Netherlands, UMCG Beatrixoord in Haren and Rehabilitation Friesland in Beetsterzwaag. A control group of sixteen healthy participants, matched by gender, age (51.38 ± 10.03 years) and handedness (Oldfield, 1971), were included. The ethical committee of the medical center of the University of Groningen approved the study protocol and a written informed consent was obtained from each participant before entering this study. The major inclusion criteria were a unilateral impaired motor function of the upper limb following stroke [wrist dorsiflexion, MRC < 5 (Gregson et al., 2000)] and stroke onset 16–52 weeks prior to participation in this study. Patients were excluded when they had a history of repeated strokes, severe cognitive dysfunction [MMSE < 24 (Folstein et al., 1975)], severe receptive aphasia (inability to understand test instructions), neglect, visual problems, neurological disorders or comorbidity which could interfere with this study. Patients had to be able to understand Dutch. Further, patients that participated in another intensive study were not able to participate in this study.

### Instruments

#### Explicit motor imagery: motor imagery questionnaire

A motor imagery questionnaire based on the MIQ-RS was administrated as an explicit motor imagery task (Gregg et al., 2010). The MIQ-RS was chosen because it includes items of functional tasks specifically aimed at hand movements (e.g., grasp a glass, push a door). Thereby, the items of the MIQ-RS correspond closely to the HLJT. The MIQ-RS questionnaire is shown to be a reliable and valid measure for motor imagery ability (Gregg et al., 2010) and is developed specifically as an instrument for the stroke population (Butler et al., 2012). Although the MIQ-RS is specifically aimed at this population, some adjustments in the protocol had to be made for administration in patients with more severe motor impairments. First, not all participants managed to completely perform the movement physically because of the variability in motor function of the upper extremity and difficulties of some patient to stand unsupported. Therefore, the test leader demonstrated the movements that had to be imagined to the participants. All participants performed the test in a sitting position. Patients as well as controls performed this task procedure in the same manner. To facilitate imagery from an internal first person perspective the examiner took place next to the participant while demonstrating the movement and participants were instructed to imagine from a first person perspective. Second, in the original MIQ-RS each action was administrated two times (from a first person visual and a kinesthetic perspective), but in our study also the non-dominant and dominant sides were assessed separately, as is suggested by Malouin et al. (2007). Adherence to the goals of the items in the questionnaire was checked by the examiner by asking how the participant imagined the action by asking what the person saw and felt.

Each item consists of the following stepwise procedure. First, the participant was asked to assume the start position: sitting on a chair with backrest and with their hands on their lap. Second, the examiner sat on a chair in front of the participant and then took place next to the participant and demonstrated a movement supported by a verbal description of that movement. Third, the experimenter took place in front of the participant and participants were asked to close their eyes and imagine either seeing or feeling as clear and vivid as possible the just demonstrated movement. In the last step of each item, the participant had to rate their experience of the ease/difficulty they could imagine the movement on an ordinal rating scale from 1 (very hard to feel/see) up to 7 (very easy to feel/see) from the visual and the kinesthetic perspective (For a detail description of the used items, instructions and protocol see Gregg et al., 2010).

#### Implicit motor imagery: computerized imagery task

We used a computerized task that consisted of three parts; a simple choice reaction time task, a visual imagery task and a HLJT. During the computer test, all participants sat in front of a laptop screen in a chair with a backrest. All participants started with the simple choice reaction time task. The visual imagery task and the HLJT were randomized between participants. In all three tasks, two types of stimuli were presented on the computer screen. The participants had to react as fast and accurate as possible on these stimuli by pressing the left or right button on a response box (Psychological Software Tools, Pittsburgh, PA). The stimulus disappeared from the screen when the participant pressed the button or when the stimulus was presented for 10 s. The responses were given with the hand of the unaffected limb. Their matched controls executed the task with the same hand regardless of their hand dominance to control for confounding effects. For each task performance was assessed by calculating the average response time (in milliseconds) and the accuracy (percentage of correct responses). Before the start of each test, the participant had the opportunity to practice the task in a block of 48 stimuli. The visual imagery task and the HLJT each contained a total 216 stimuli, divided in three blocks of 72 stimuli with a short break period between the blocks.

***Simple choice reaction time task***. A simple choice reaction time task was included to control whether simple reaction time was affected in stroke patients. The HLJT is based on time isochrony and therefore it is important to control whether a latency in reaction time on the HLJT is the result of impaired motor imagery ability instead of a more general impaired reaction time. The participants had to react to two types of presented stimuli, an “O” or “X,” by pressing respectively the left or right button on a response box.

***Hand laterality judgment task***. Implicit motor imagery was assessed with a HLJT (Parsons, 1987). Participants had to determine the laterality of a rotated hand presented on the computer screen. The hands were shown from the backside or palm side of the hand. The stimuli of presented hands were rotated at six different angles, covering the full circle at a spacing of 60° (0, 60, 120, 180, 240, 300°). The orientation of the hand with fingers pointing upward was defined as an angle of 0°. Participants had to react to these stimuli by pressing the button (left or right) corresponding to the laterality of the hand. The participants were not allowed to rotate their hand or look at their own hand during this task. Adherence to this task procedure was carefully controlled by the experiment leader, who was present during the whole experiment.

***Visual imagery task***. The visual imagery task (de Lange et al., 2005) was included in this study to control whether the participants performance on the HLJT was related to visual imagery ability. Stimuli of mirror-reversed and regular capital alphabetic characters, “R” and “F,” were presented on the computer screen. The characters were rotated at the same angles (0, 60, 120, 180, 240, 300°) as stimuli in HLJT. The orientation when the “R” or “F” was upright was described as an angle of 0°. Participants had to decide as fast and accurate as possible whether the letter was a mirror-reversed or a normal letter by pressing respectively the left or right button on the response box.

#### Brunnström fugl-meyer scale

The arm-hand function of the affected limb was administrated with the Brunnström Fugl-Meyer scale (BFM) (Fuglmeyer et al., 1975). The Fugl-Meyer scale is shown to be a reliable and valid tool for the evaluation of motor recovery in stroke patients (Gladstone et al., 2002). This test is used in clinical settings for determining reflexes, movement, coordination and speed of the upper extremity. Each item could be scored with 0–2 points on an ordinal scale (0 = no movement possible; 1 = impaired movements possible; 2 = movements possible). A total score was inside a range of 0–60 points, because reflexes were not examined in this study.

#### Utrecht arm/hand test

The Utrecht Arm/hand Test (UAT) is a clinical measure to obtain the arm-hand function of patients (van Reenen et al., 2001). The items of this test are scored on an ordinal scale where eight items represent the following function of the upper limb: a-functional arm (score 0); flexion-synergy (score 1), first distal selectivity (score 2), wrist dorsal flexion (score 3), hook grip (score 4), cylinder grasp (score 5), tweezers grasp (score 6), and clumsy hand (score 7).

### Procedure

The testing procedure was similar for all participants. The assessment took place in a quiet room in one of the rehabilitation centers or at home. The measurement started with the computerized imagery tasks. This was followed by the examination of the motor function. Finally, the MIQ-RS questionnaire was completed by the participants.

### Data analysis

For the computer task, mean reaction times of correct responses and mean accuracy scores of responses with a latency between 350 and 10.00 ms were calculated and included in the analysis. First analyses revealed no differences between stimuli or items of the affected or the non-affected limb in patients and therefore left and right stimuli were grouped together for both the questionnaire as well as for the computer task. First a reliability analysis was done for the visual and kinesthetic component of the adapted MIQ-RS. Then total mean scores were calculated for the visual and kinesthetic components of the adapted MIQ-RS. In accordance with Page et al. (2001), who used a cut-off of 25 (on a maximum of 56 on the original MIQ-R scale), a mean score on one of the subscales below section Differences in Imagery Ability Between Patients and Controls was considered as low imagery ability. Because of non-normal distributed data, non-parametric tests were used for analysis. The difference between the control and the patient group was determined with a Mann-Whitney U test, with α < 0.05. To control whether individual patients differed significantly on the computer task from the control group, a modified *T*-test with α < 0.05 was used (Crawford and Howell, 1998). The relationship between the MIQ-RS, HLJT, visual imagery task, BFM, and UAT was calculated with a Spearman correlation coefficient. Finally, to determine which strategies were used by the patients and the control group, separate repeated measures ANOVAs were performed on the RTs for the HLJT and the visual imagery task. To determine whether participants still use a motor strategy in solving a motor imagery task the stimuli were collapsed in sets of comfortable (medial) and awkward (lateral) orientations resulting in two within-participants factors for the ANOVA: biomechanical orientation (awkward, comfortable) and rotation (60, 120°), and with group (patients, controls) as a between-subjects factor. A corresponding ANOVA setup was also used for the visual imagery task with alphanumeric character orientation (clockwise, anticlockwise) and rotation (60, 120°) as within-subject factors, and with group (patients, controls) as a between-subjects factor. All data were analyzed with IBM SPSS version 17.0.

## Results

### Internal reliability

Cronbach's alpha for the visual motor imagery subscale of the MIQ-RS was high with α = 0.98 for the patient group, and α = 0.95 for the control group. Cronbach's alpha for the kinesthetic motor imagery subscale of the MIQ-RS was also high with α = 0.98 for the patient group, and α = 0.98 for the control group.

### Differences in imagery ability between patients and controls

#### Patient group vs. control group

Table 1 shows the results on the questionnaire and HLJT for the group of patients and the group of controls. Patients scored significantly lower on both, the visual motor imagery component (*U* = 60; *p* = 0.010) and the kinesthetic motor imagery component (*U* = 63.5; *p* = 0.015) of the MIQ-RS compared to controls. In the simple choice reaction time task, the reaction time did not differ significantly between the group of patients and controls (*U* = 24; *p* = 0.200). Also the accuracy on this task was similar for both groups (*U* = 105.5; *p* = 0.355). The patients reacted slower on the visual imagery task (*U* = 68; *p* = 0.024) as well as on HLJT (*U* = 58; *p* = 0.008) compared to the control group. No significant differences between patients and controls were found on the visual imagery accuracy score (*U* = 93.5; *p* = 0.189) and the accuracy score on the HLJT (*U* = 111.5; *p* = 0.532).

**Table 1:** **Scores (mean/*SD*) on the MIQ-RS and hand laterality task, the object identification task and the visual imagery task for the group of controls and patients and the individual patient scores for the UAT and BFM (*N* = 16)**.

	**MIQ-RS**		**SCT**		**VIT**		**HLJT**		**UAT**	**BFM**
	**V**	**K**	**RT**	**ACC**	**RT**	**ACC**	**RT**	**ACC**		
Controls	5.9 (1.0)	4.9 (2.0)	479 ± 86	98 ± 3	903 ± 249	97 ± 3	1704 ± 564	93 ± 5		
Patients	4.0 (2.1)^*^	3.1(2.1)^*^	523 ± 95	98 ± 2	1118 ± 227^*^	94 ± 7	2568 ± 902^*^	89 ± 1		
1	7.0	7.0	470	96	1275	100	4873^*^	94	7	60
2	1.1+	1.0+	698^*^	100	1574^*^	93	3322^*^	93	3	31
3	2.9+	1.6+	579	96	830	99	2368	87	6	59
4	1.0+	1.0+	378	98	981	96	1560	96	7	59
5	4.9	1.7+	554	100	1493^*^	93	1827	39^*^	7	60
6	4.8	5.1	494	96	1301	96	2525	93	7	55
7	4.4	4.0	685^*^	98	1034	89^*^	1866	91	7	58
8	6.3	5.2	496	96	968	77^*^	2462	78^*^	7	60
9	5.4	5.4	540	96	1120	83^*^	3414^*^	81^*^	6	35
10	1.9+	1.0+	563	98	1194	88^*^	1632	99	0	0
11	6.2	5.8	445	98	799	99	1505	98	7	58
12	3.6	3.6	421	100	863	100	2565	94	0	2
13	6.0	1.0+	658	100	1074	98	2560	98	4	34
14	1.6+	3.4	470	100	964	100	2311	94	6	55
15	1.1+	1.2+	421	98	1303	97	3755^*^	97	7	59
16	6	2.0+	500	100	1112	94	2536	92	7	60

SCT, Simple choice task; VIT, Visual Imagery Task; HLJT, Hand Laterality Judgment Task; V, visual motor imagery component (score); K, kinesthetic imagery component (score); RT, reaction time (ms); ACC, accuracy (% correct responses).

* Significantly different compared to the control group.

+ Below the cut-off score.

#### Individual patients vs. control group

The individual score of patients on the questionnaire, hand laterality task and the BFM and UAT are shown in Table 1. Six out of the 16 patients (37.5%) scored below the cut-off on the visual imagery component of the questionnaire indicating impaired visual motor imagery ability. Eight patients (50%) scored below the cut-off on the kinesthetic imagery component of the questionnaire indicating impaired kinesthetic imagery ability. Three patients scored lower on the kinesthetic component without scoring lower on the visual component of the MIQ-RS (patients 5, 13, and 16). One patient, patient 14 scored lower on the visual component of the motor imagery scale without scoring lower on the kinesthetic component. A significantly lower reaction time than the control group was found for two patients in the simple choice reaction time task, two patients in the visual motor imagery task, and three patients in the HLJT. Patient 2 (see Table 1) scored significantly below the mean reaction time of the control group on all three tasks. Two of the 16 patients had significantly lower accuracy scores both on the visual imagery task and HLJT. Two patients showed less accurate responses independently on the visual imagery task and one patient only on the HLJT. Only one of the patients with low accuracy scores on one of the computerized imagery tasks scored at around chance level (patient 5). All other patients scored well above chance on the computerized imagery tasks.

### Correlation between the questionnaire, the hand laterality task and the visual imagery task

The Spearman correlation coefficients between MIQ-RS and the computerized imagery task for the groups of controls and patients are respectively shown in Tables 2, 3. No significant correlation was found between the scores on the components of questionnaire and hand laterality task or visual imagery task for patients or controls. A significant positive correlation between the visual and kinesthetic motor imagery component of the questionnaire was found in both groups. This was the only significant correlation between imagery measures in patients. There were no significant correlations between measures for implicit and explicit motor imagery in both groups. Table 2 shows, for the control group, a high significant positive correlation between the accuracy of the HLJT and visual imagery task. The positive correlation between the reaction time of implicit HLJT and the visual imagery task was also significant in the control group. The reaction time on the visual imagery task was negatively correlated with the accuracy on the same task in the control group. No significant correlation was found between the UAT and BFM and the different types of imagery. The UAT and BFM did correlate significantly with each other.

**Table 2:** **Spearman correlations between the questionnaire, hand laterality task visual imagery task of controls (*N* = 16)**.

		**Questionnaire**		**Hand laterality task**		**Visual imagery task**	
		**V**	**K**	**RT**	**ACC**	**RT**	**ACC**
Questionnaire	V	1					
	K	0.50^*^	1				
Hand laterality task	RT	0.00	−0.13	1			
	ACC	0.35	0.26	−0.27	1		
Visual imagery task	RT	−0.15	−0.06	0.86^**^	−0.48	1	
	ACC	0.32	0.20	−0.37	0.67^**^	−0.53^*^	1

V, visual motor imagery component (score); K, kinesthetic imagery component (score); RT, reaction time; ACC, accuracy.

* p < 0.05

** p <0.01.

**Table 3:** **Spearman correlations between the questionnaire, the hand laterality task and the visual imagery task of patients (*N* = 16)**.

		**Questionnaire**		**Hand laterality task**		**Visual imagery task**		**Motor function**	
		**V**	**K**	**RT**	**ACC**	**RT**	**ACC**	**BFM**	**UAT**
Questionnaire	V	1							
	K	0.69^**^	1						
Hand laterality task	RT	0.14	0.15	1					
	ACC	−0.23	−0.32	−0.10	1				
Visual imagery task	RT	−0.13	−0.25	0.42	−0.11	1			
	ACC	−0.04	0.10	0.14	0.43	−0.37	1		
Motor function	BFM	0.38	0.30	−0.04	−0.45	0.02	−0.02	1	
	UAT	0.33	0.42	−0.16	−0.27	0.08	−0.09	0.83^**^	1

V, visual motor imagery component (score); K, kinesthetic imagery component (score); RT, reaction time (ms); ACC, accuracy % correct responses).

** p < 0.01.

### Reaction time analysis

A repeated measures ANOVA on the reaction time scores of the HLJT showed a main effect of orientation, with *F*_(1, 30)_ = 13, 78, *p* < 0.01, rotation, with *F*_(1, 30)_ = 40, 24, *p* < 0.001 and a significant interaction between orientation and group, with *F*_(1, 30)_ = 5, 58, *p* < 0.05, rotation and group, with *F*_(1, 30)_ = 7, 62, *p* < 0.05 and between orientation and rotation, with *F*_(1, 30)_ = 4, 20, *p* = 0.05. *Post-hoc* analysis showed that patients were significantly slower than controls on lateral, more awkward, orientated stimuli of hands than on medial orientations with the same extend of rotation. Analysis of the reaction times of the visual imagery task only showed a main effect of rotation with *F*_(1, 30)_ = 96, 13, *p* < 0.001. No further main or interaction effects were found for the visual imagery task.

## Discussion

With this study we wanted to explore the prevalence of implicit and explicit motor imagery ability impairments and investigate the relationship between implicit and explicit motor imagery ability measures in stroke patients. Patients in this study scored significantly below controls on both the visual and the kinesthetic component of the adapted MIQ-RS. However, they did not differ significantly from the control group on the accuracy scores of the HLJT or the visual imagery task. More importantly, our results showed that there is discrepancy between performance of stroke patients on the explicit and implicit motor imagery tasks. The results from this study showed no significant correlations between the HLJT and the MIQ-RS. Neither in stroke patients, nor in age matched controls, a significant correlation between implicit and self-reported explicit motor imagery ability was found. To our knowledge this the first time that a divergence between results on a phenomenological explicit motor imagery measure and an implicit motor imagery measure is shown in stroke patients.

Our results show that the subjective experience of the ease of imagining a movement does not have to be related to implicit motor imagery ability after stroke. These results are in line with the model of overt and covert action simulation and the distinction therein between explicit and implicit motor imagery proposed by Jeannerod (2001). The results are also in close accordance with the study of McAvinue and Robertson (2007) where also no correlation between implicit and explicit motor imagery was found. However, in the study of McAvinue and Robertson (2007) only healthy adults were included. In our study we extend these results by showing that also in stroke patients no correlation is found between the phenomenological experience of motor imagery and the performance on implicit motor imagery ability. In the light of the recent advocacy for screening for motor imagery ability in motor-imagery based rehabilitation programs (Jackson et al., 2001; Braun et al., 2006, 2013 in this issue; de Vries and Mulder, 2007; Simmons et al., 2008) these results have an important clinical implication. Our study shows that by selecting patients on the basis of subjective reports only, researchers could risk excluding patients that might still have intact motor imagery ability. Therefore, a screening procedure where different imagery measures are used seems more appropriate for the use of screening stroke patients in motor-imagery based rehabilitation programs.

We used the MIQ-RS as a measure for the vividness of explicit motor imagery ability. We chose the MIQ-RS specifically because of the items in this questionnaire focus on arm and hand movements, thereby maximizing the relationship with the HLJT. However, although the MIQ-RS has shown to be a reliable and valid measure for motor imagery ability (Gregg et al., 2010) and is also developed specifically for use in the stroke population (Butler et al., 2012) we did make some adjustments to the protocol that could have influenced our results. Because not all stroke patients were able to complete all movements physically, all participants had to watch a demonstration of the intended movement instead of performing the movement themselves. Therefore, we cannot be sure that all participants did indeed use an internal motor imagery strategy. It could be that by observing a demonstration of the movements our participants were more inclined to imagine movements from an external perspective.

Indeed, a recent study (Williams et al., 2011) showed that observation of movements could facilitate the ease with which movements are imagined. In their study observation of a movement shortly before a participant had to imagine a movement led to higher MIQ scores only when the perspective of the observed and to be imagined movement were congruent with each other. Hence, it could be that in our set-up with the experiment leader sitting next to the participant that participants were more inclined to use an external perspective and as such may have experienced more difficulty imagining the movement from the first person perspective. However, the test conditions were the same for control participants. Therefore, we believe this could hardly explain the differences found on the MIQ-RS in our study. For follow up studies, to better control for these issues, it would be better to use the MIQ3. The MIQ-RS, as is also pointed out by Roberts et al. (2008), cannot distinguish between the internal and an external visual motor imagery perspective. In the new version of the MIQ-RS, the MIQ3 (Williams et al., 2012), external, internal and kinesthetic components are assessed separately.

Williams et al. (2011) also showed that motor experience could influence how easily a movement is imagined. Although, in our study we controlled for this by following the same protocol for control participants and patients it could be that differences in motor experience still had an influence on the ease with which movements could be imagined. Control participants, unlike patients, clearly have had more chance at performing these movements more recently and more frequently than stroke patients. This could have resulted in lower scores in stroke patients compared to controls. However, our scores were similar to that of other studies. We showed a high internal reliability, comparable to those of the original questionnaire (Gregg et al., 2010). Moreover, we did see a difference between the visual and kinesthetic scale, the visual imagery score was higher than the kinesthetic imagery score, both in stroke patients as well as in healthy controls. These results are comparable to studies with the original MIQ-RS and point to a certain degree of sensitivity to dissociate between visual and kinesthetic imagery ability (Butler et al., 2012). Also, a study by Confalonieri et al. (2012) showed comparable scores to our results in a neuroimaging study of stroke patients where the MIQ-RS was also used.

It could be that our patients adopted different strategies than controls for solving the HLJT. For instance, a recent study with stroke patients by Daprati et al. (2010) showed that patients can in some cases adopt alternative strategies, for example use a visual imagery strategy, for solving the HLJT. However, in our study patients, like controls, showed longer reaction times for biomechanical awkward stimuli and this effect was not seen on the reaction time distributions of the visual imagery task. The longer reaction times for more awkward orientations indicate that patients and controls employed the same strategy in the HLJT and that indeed implicit motor imagery was used.

Interestingly, when looking at the individual patients our results are somewhat heterogeneous. Seven patients reported that it was hard to feel the imagined actions whereas scoring well above chance on the HLJT. Five of these patients also found it hard to imagine seeing the imagined action indicating simultaneous impairment of visual and kinesthetic motor imagery. However, one patient (patient 5) also showed an interesting pattern of results. This patient was selectively impaired on the kinesthetic component of the adapted MIQ-RS. Moreover, this patient scored at around chance level on the HLJT. This patient's performance on the HLJT could not be explained by mental slowing because performance on the simple choice task was normal. This patient also scored well above chance on the visual imagery task, suggesting intact visual-spatial capacity. The fact that his kinesthetic ability was selectively below the cut-off score simultaneously with a selective impairment on the HLJT without showing other imagery deficits suggests that to some extent correspondence between conscious experience and implicit ability of motor imagery is also possible after stroke.

At the same time the heterogeneous results in our patients show that the assessment of motor imagery ability in stroke patients is a complex task. The patients that found it very hard to imagine movements might not be able to benefit from mental practice because of the emphasis in mental practice on wilful conscious modulation of motor imagery. On the other hand, this might suggest that these patients, because of their intact implicit motor imagery ability, might still benefit from probing the action system covertly, for instance by observing actions or implicit learning. Given the recent research results on the differences between implicit and explicit learning (see Dew and Cabeza, 2011 for a discussion) makes this question certainly seem worthwhile to explore in more detail. In this respect Holmes and Calmels (2008) have reviewed potential problems for motor imagery based mental practice in the sport setting and have suggested that with an observation based approach some aspects of the covert use of our action system can be better controlled, a direction possibly also worth exploring further in the domain of stroke.

Most motor imagery instruments are originally developed en validated in young healthy populations. For instance, although the original developers of the MIQ-RS adapted the instrument for use in a rehabilitative setting (Butler et al., 2012), there are only a few studies reporting results using the new MIQ-RS with stroke patients. Stroke patient are a far more heterogeneous population than the healthy young adults and this makes interpretation of the results of motor imagery instruments in stroke patients difficult. This is particularly important because in stroke patients the severity and extend of the hemiparesis can be complicated by the presence of neuropsychological deficits in these patients. Deficits in working memory, apraxia, depression, motivational problems, (motor) neglect and anosognosia are all know in some instances to complicate the hemiparesis (Gialanella and Mattioli, 1992; Paolucci et al., 1996; Pohjasvaara et al., 2002; Malouin et al., 2004) and it is likely that the same is true for performance on motor imagery measures, explicit and implicit.

For instance Malouin et al. (2004) showed that performance of motor imagery practice is related to working memory capacity. Furthermore, recent studies on neglect after stroke show a more differentiated picture of different forms and types of neglect. For instance, motor neglect is often under recognized but can influence motor performance (Punt and Riddoch, 2006) and patients can have neglect selectively for near space (peripersonal) as well as for far space (extrapersonal) and for specific modalities (see Halligan et al., 2003 for a discussion). The notion of neglect affecting extrapersonal and peripersonal space differently is akin to the dissociation between first person and third person imagery. A limitation of our study is that we screened patients for neglect, however, we did not control for all the different types of neglect leaving open the possibility that more specific neglect types were undetected and influenced the patients test outcome. In the same regard, other specific neuropsychological deficits could influence motor imagery test performance and the possibilities for and adherence with mental practice regimes. Future studies that systematically study the relationship of neuropsychological disorders with motor imagery ability could greatly enhance our knowledge in this respect.

Another limitation of our study was that we only used two measures for motor imagery. By including other type of measures, such as mental chronometry tasks, prospective action judgment task and also including phenomenological self-reports that are aimed at measuring other subjective components of the imagery process we could be able to further our understanding of how the different components of motor imagery ability relate to each other. A better understanding of the type of impairment of the action system by using different methods and instruments ranging from implicit to explicit, self-rating, physiological, neuropsychological and chronometric instruments, could potentially lead to a better differentiated treatment tuned to the patients characteristics in particular.

Conclusively, our study shows that in stroke patients implicit and explicit motor imagery can be differently affected. The subjective experience of the ease of imagining a movement does not have to be related to implicit motor imagery ability after stroke. Given the recent advocacy for the use of screening instruments to assess motor imagery ability of patients before they enter rehabilitation programs (Braun et al., 2006, 2013 in this issue; Malouin et al., 2013 in this issue) these results have an important clinical application and suggest that screening procedures based solely on subjective instruments could risk excluding patients whose motor imagery ability might still be intact. Our results articulate the complex relationship between the phenomenological conscious experience of motor imagery and use of motor imagery in individual patients and caution the use of one tool as an instrument for use in screening, selecting and monitoring stroke patients.

### Conflict of interest statement

The authors declare that the research was conducted in the absence of any commercial or financial relationships that could be construed as a potential conflict of interest.
